# Impact of COVID-19 research: a study on predicting influential scholarly documents using machine learning and a domain-independent knowledge graph

**DOI:** 10.1186/s13326-023-00298-4

**Published:** 2023-11-28

**Authors:** Gollam Rabby, Jennifer D’Souza, Allard Oelen, Lucie Dvorackova, Vojtěch Svátek, Sören Auer

**Affiliations:** 1 L3S Research Center, Leibniz University Hannover, Hanover, Germany; 2 Department of Information and Knowledge Engineering, Prague University of Economics and Business, nám. Winstona Churchilla 1938/4, 120 00 Prague, Czech Republic; 3 Leibniz Information Centre for Science and Technology, Hannover, Germany; 4 Department of Econometrics, Prague University of Economics and Business, Prague, Czech Republic

**Keywords:** Influential scholarly document prediction, Machine learning algorithms, Text mining, COVID-19, World health organization, Domain-independent knowledge graph

## Abstract

Multiple studies have investigated bibliometric features and uncategorized scholarly documents for the influential scholarly document prediction task. In this paper, we describe our work that attempts to go beyond bibliometric metadata to predict influential scholarly documents. Furthermore, this work also examines the influential scholarly document prediction task over categorized scholarly documents. We also introduce a new approach to enhance the document representation method with a domain-independent knowledge graph to find the influential scholarly document using categorized scholarly content. As the input collection, we use the WHO corpus with scholarly documents on the theme of COVID-19. This study examines different document representation methods for machine learning, including TF-IDF, BOW, and embedding-based language models (BERT). The TF-IDF document representation method works better than others. From various machine learning methods tested, logistic regression outperformed the other for scholarly document category classification, and the random forest algorithm obtained the best results for influential scholarly document prediction, with the help of a domain-independent knowledge graph, specifically DBpedia, to enhance the document representation method for predicting influential scholarly documents with categorical scholarly content. In this case, our study combines state-of-the-art machine learning methods with the BOW document representation method. We also enhance the BOW document representation with the direct type (RDF type) and unqualified relation from DBpedia. From this experiment, we did not find any impact of the enhanced document representation for the scholarly document category classification. We found an effect in the influential scholarly document prediction with categorical data.

## Introduction

Text classification is a fundamental task in natural language processing, with applications in various down-stream tasks, including sentiment analysis [1, 2], named entity recognition [3, 4], and part-of-speech tagging [5]. It can be applied to various granularities of text, such as words, phrases, sentences, snippets, and articles. The classification or prediction can be performed using machine learning-based methods, such as supervised, semi-supervised, or unsupervised.

This research focuses on predicting influential scholarly documents using short texts such as abstracts. Predicting the impact of scholarly documents is essential for understanding the scientific areas they belong to and organizing them more effectively. In the context of influential scholarly document evaluation [6], we emphasize the importance of aligning quantitative metrics for the specific research domain such as COVID-19, even though institutional data was not used. While we acknowledge potential language bias due to the dominance of English in COVID-19-related scholarly documents, our study primarily focuses on worldwide publications. Transparency and predefined rules are crucial for robust evaluation, supported by a browser extension for accuracy verification by domain experts. Citation practices across fields were not a significant factor in our study confined to the biomedical domain. Our approach avoids undue precision, recognizing the inherent variability in citation counts. Considering the systemic effects of indicators, which should be anticipated and addressed through a comprehensive suite of indicators, is essential. Our experiment utilized textual or contextual elements such as titles, keywords, abstracts, citations, and many more. In most cases, the full texts of scholarly documents are private and are computationally expensive to process.

## Research questions

This research was organized into two main parts. The first part deals with predicting influential scholarly documents based on scholarly document abstracts. In contrast, the second part focuses on understanding the impact of leveraging a domain-independent knowledge graph for influential scholarly document prediction. In this section, we outline the three research questions (RQ) that underlie our work. RQ1 and RQ2 tackle influence prediction on two variants, a semantically categorized and an uncategorized dataset, of the COVID-19-themed task-specific dataset. RQ3 in addition incorporates the consideration of a knowledge graph in the prediction task.

- **RQ1**: Which machine learning classification method works best for scholarly document category classification?
- **RQ2**: How does using an uncategorized versus a categorized corpus affect the classification of influential scholarly documents when each machine learning method is run separately for each category?
- **RQ3**: What is the impact of leveraging a domain-independent knowledge graph, specifically DBpedia, in the predictive task of determining influential scholarly documents in COVID-19 research?

## Related work

Traditional machine learning methods, such as random forest [7], k-nearest neighbors [8], naive bayes [9], and many more have been widely used for various classification tasks. However, these methods require a balanced dataset to perform well in per-class prediction accuracy and overly coarse classifications for domain-independent corpora. Recent research has shown that these methods perform well for domain-dependent tasks, particularly in classifying bio-medical scholarly documents [10]. Deep neural networks [11], such as convolutional and recurrent networks [12], have also been applied to classification tasks. Additionally, pre-trained language models, such as BERT [13], are increasingly used for deep learning-based techniques.

Various document representation methods have been proposed for classification or prediction tasks. Distributed dense vector representations of text, such as word embeddings [14], have been developed and used to measure semantic relationships between words. Word embeddings have also been applied to similarity analysis and semantic parsing [15]. Other embeddings have also been developed, such as character-level, phrase, and sentence embeddings [16, 17]. The bag of words (BoW) [18], and term frequency-inverse document frequency (TF-IDF) [19] are commonly used text representations for classification tasks. BoW does not consider syntactical or sequential information but represents text as a collection of unordered word-level tokens. The BoW has been integrated with linguistic, grammatical, and structural aspects in classifying phrases in scholarly document abstracts [20].

This study focuses primarily on the contextual part of scholarly documents, specifically the abstracts, and examines the effect of using an external knowledge graph (KG) for classification. A KG is a common framework for representation based on analyzing and extracting entities and relationships from structured and unstructured data. KGs are a practical technique for enhancing knowledge and have been compared with existing ones such as DBpedia [21]. The study found that using KGs could increase the accuracy of predictions by enhancing entities from abstracts and representing short texts with semantically similar texts in a similar context without common words. While domain-independent KGs could provide irrelevant features. In particular, KGs provide explicit, unambiguous, and complementary domain-independent or domain-specific knowledge. The study also compared other document representation methods, such as the bag of words, extensively used for the scholarly document classification task.

Citation counts of new scholarly documents are often only available after a while, especially in niche fields where the citations may take a considerable time to accrue. The citation count is an important metric used to assess the impact and influence of a scholarly document within a research community. Without immediate citation counts, a classifier that can identify influential scholarly documents can provide valuable insight to researchers and help guide future research directions. Such a classifier would need to consider other factors that contribute to a scholarly document’s influence, such as the research content, the journal in which the scholarly document is published, the relevance of the scholarly document’s topic to current research, and many more. Developing a classifier that can effectively identify influential scholarly documents without relying on citation counts can provide a valuable tool for the scientific community.

**Fig. 1:**
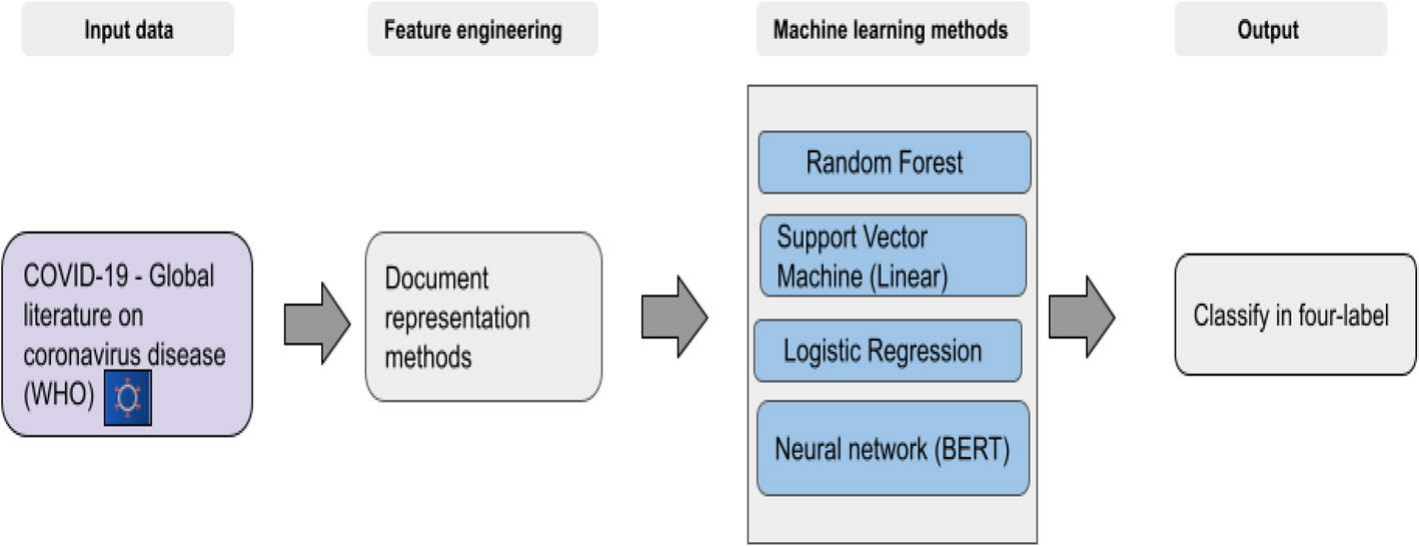
Overview of the scholarly documents label (WHO-4) classification workflow

**Fig. 2:**
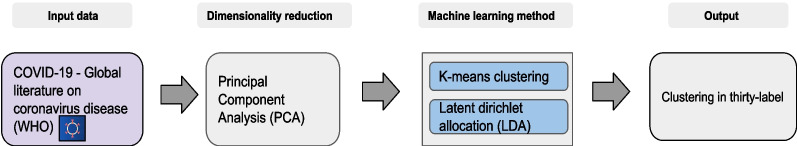
Overview of the workflow for the scholarly document’s thirty-label categorization

**Fig. 3:**
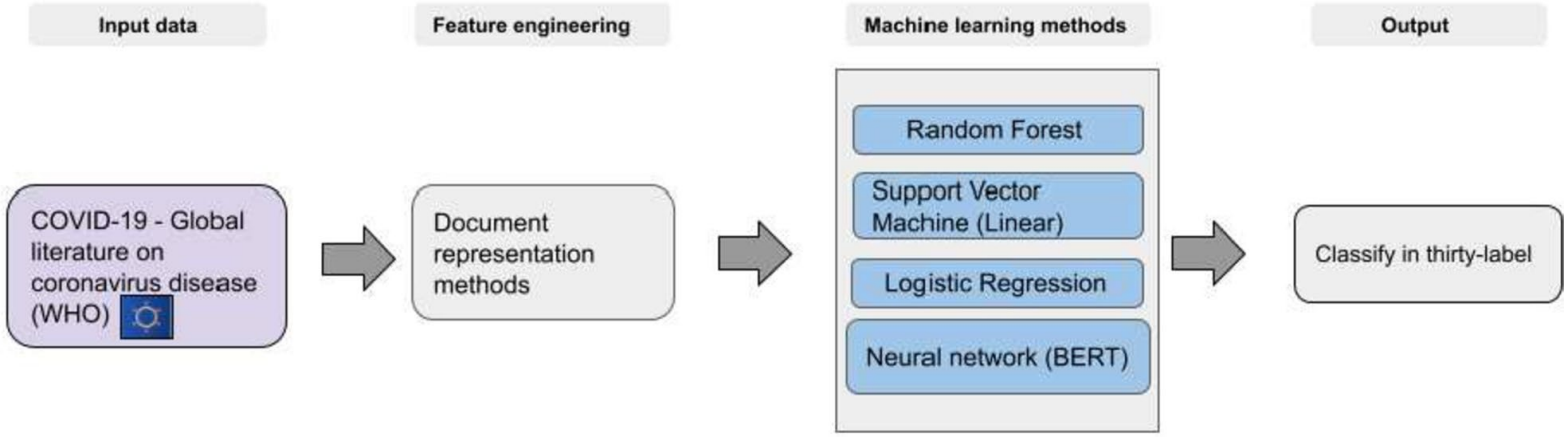
Overview of the scholarly documents label (WHO-30) classification workflow

**Fig. 4:**
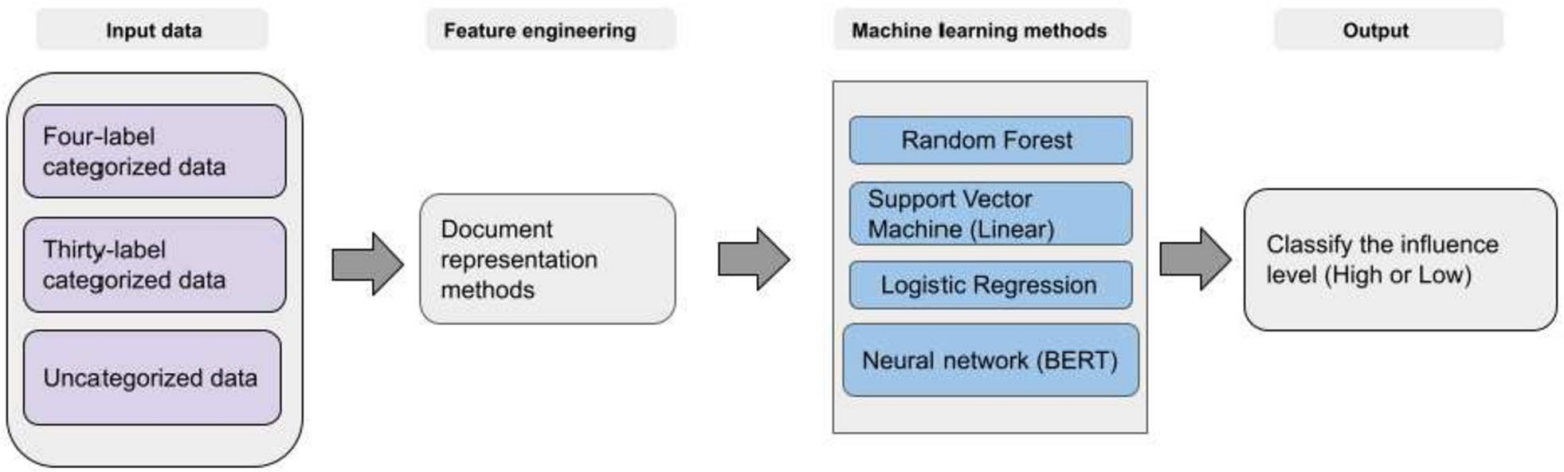
Overview of the workflow for classifying the influential scholarly documents prediction

## Methods

### Predicting influential scholarly documents

One of the primary objectives of this workflow was to identify influential scholarly documents within different categories and uncategorized data. The World Health Organization (WHO) assigned the used corpus into four categories of data that may need to be sufficiently granular; we divided the dataset into thirty categories. This categorization aimed to evaluate the impact of more categorized data on identifying influential scholarly documents. The classification workflow was executed in two experimental setups to achieve this goal. As depicted in Fig. 1, the first experiment utilized a corpus of four labelled categories, which the World Health Organization verified. The second experiment employed a corpus of thirty labelled categories, utilizing a machine learning-based clustering method. The workflow for the scholarly document’s thirty labels categorization is presented in Fig. 2. In Fig. 3, the experiment utilized a corpus of thirty labelled categories, which we developed by utilizing a categorization method (Fig. 2). In Fig. 4, the four labels, thirty labels, and uncategorized data were considered input for the influential scholarly document prediction. The impact of categorized data was compared with uncategorized data on the classification task with different machine learning-based methods.

**Table 1:** Label count (used for this experiment) of the World Health Organization (WHO) (COVID-19 Global literature on coronavirus disease) corpus

Label	Label count
**Vaccines**	33465
**Long_Covid**	27937
**Traditional_medicine**	5487
**Variants**	17331
**Total**	84220

#### Input corpus

One of the primary objectives of this workflow was to identify influential scholarly documents within different categories and uncategorized data. The World Health Organization (WHO) assigned articles in the used corpus into four categorized data, which may not be sufficiently granular; we further divided the dataset into thirty categories. This categorization aimed to evaluate the impact of more categorized data on identifying influential scholarly documents. The classification workflow was executed in two experimental setups to achieve this goal. As depicted in Fig. 1, the first experiment utilized a corpus of four labelled categories, which the World Health Organization verified. The second experiment employed a corpus of thirty labelled categories, utilizing a machine learning-based clustering method. The workflow for the scholarly document’s thirty labels categorization is presented in Fig. 2. In Fig. 3, the experiment utilized a corpus of thirty labelled categories, which we developed by utilizing a categorization method (Fig. 2). In Fig. 4, the four labels, thirty labels, and uncategorized data were considered input for the influential scholarly document prediction. The impact of categorized data was compared with uncategorized data on the classification task with different machine learning-based methods.

The primary data source for this work is the World Health Organization (WHO) COVID-19 corpus^1^ containing scholarly documents mostly from biology and medicine applicable to the COVID-19 crisis (Table 1). The WHO COVID-19 corpus was enriched with citation counts from the OpenCitations corpus^2^ by queries based on the respective scholarly document DOIs. The four values of the target variable (“Topics” according to the WHO) were utilized for the multi-class classification task, which the WHO verifies, and the topic modeling extracted the thirty different target variables. For the influential scholarly documents classification, the target variable for the binary classification was derived from citation counts, which are not part of the corpus and were obtained using the median value as a threshold.

For the scholarly document classification task, we enhanced the dataset by adding different levels of categorization. The “WHO-4” dataset was enhanced with four different target classes (Vaccines, Long_Covid, Traditional_medicine, and Variants), which the WHO verified. On the other hand, the “WHO-30” dataset was enhanced with thirty different target classes, which were obtained through a machine learning-based clustering method.

In the next phase of this study, we aim to investigate the effect of utilizing uncategorized versus categorized corpus on the classification of influential scholarly documents. To accomplish this, a sample of 50000 and 1906 scholarly documents about COVID-19, published in 2022 and 2023, were randomly selected from the World Health Organization’s database. However, a subset of 8080 and 69 scholarly documents was removed from the sample due to the absence of abstracts.

**Fig. 5:**
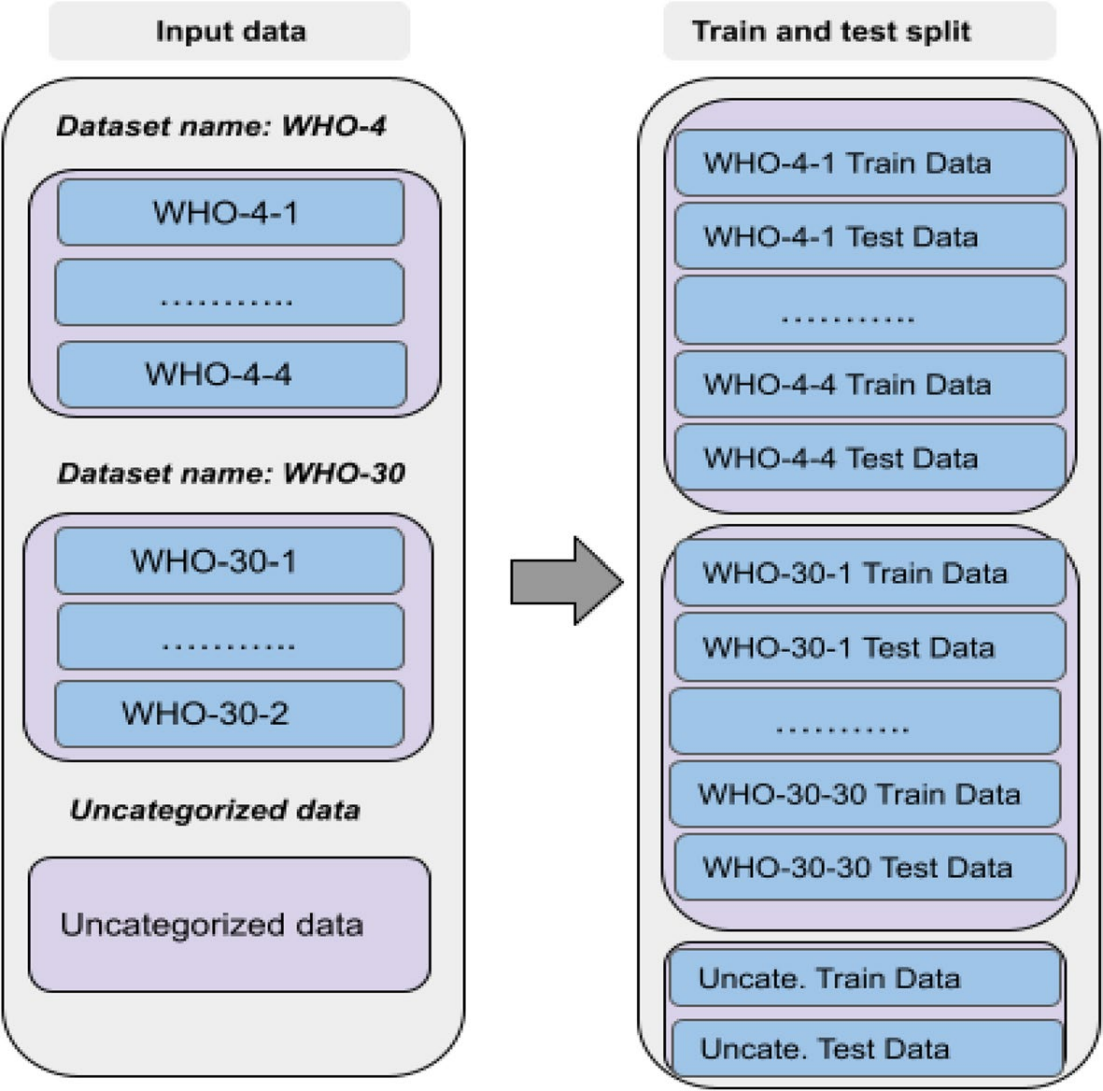
Elbow curve for choosing the number of clusters

**Table 2:** Example terms for each cluster for 5 example classes (from 30)

Cluster	Terms	Cluster name
1	dose, variants, children, media, model, community, intention, unvaccinated, status, low.	dose_variants _children
2	booster, cases, omicron, hospital, immunity, primary, fully, controls, chadox, individuals.	booster_cases _omicron
3	coronavirus, tcm, viral, mrna, million, world, cases, antibodies, plants, cancer.	coronavirus_tcm _viral
4	treatment, levels, score, groups, factors, cancer, iqr, risk, death, yards.	treatment_levels _score
5	cytokine, neurological, ill, storm, liver, lung, viral, risk, care, vitamin.	cytokine _neurological_ill

#### Machine learning methods (clustering and topic modeling)

To categorize the WHO dataset into thirty different categories (which we referred to as the WHO-30 dataset), we employed the k-means clustering [22] method. The k-means clustering algorithm is widely used for grouping similar data points based on their features. Additionally, we applied the technique of Principle Component Analysis (PCA) [23] to reduce the dimensionality of the data. We utilize the n_components^3^ parameter and set the principal components to keep. In this case, we have set it to 0.95, which means the method will keep the number of principal components needed to explain 95% of the variance. The PCA method is used to identify patterns in data and reduce the number of features while maintaining important information. A random state of 42 was used to initialize the PCA algorithm to ensure the reproducibility of the results. We utilized TF-IDF vectorization from the scikit-learn library to vectorize the data. We employed k-means on the vectorized data to cluster the scholarly documents. To determine the optimal number of clusters, k, we utilized the Elbow Method [24]. This method involves computing the sum of squared distances from each point to its assigned center. In Fig. 5, we present the elbow curve for choosing the number of clusters.

**Fig. 6:**
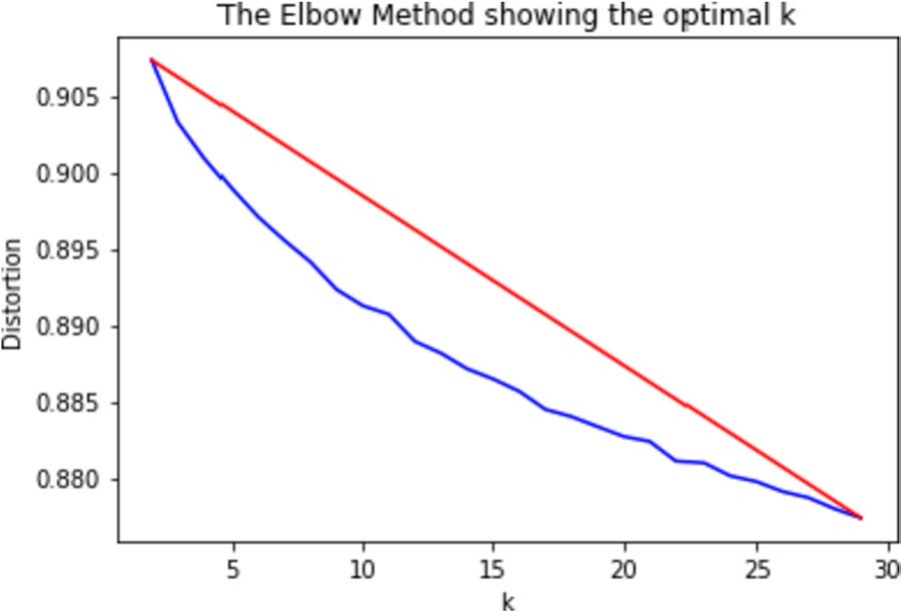
Overview of the workflow for the test and train split of the WHO-4 and WHO-30 data. The number in the dataset name, e.g., number 3 in WHO-4-3, is the id of the label within the dataset here three would correspond to the WHO-4 label $Traditional\_medicine$

We applied clustering to the entire WHO corpus of scholarly documents and identified the essential keywords in each cluster. We used k-means clustering to group the documents and topic modeling to identify the themes within each cluster. To efficiently identify the themes, we provided keywords to explore the possibilities within each cluster. To discover topics of the scholarly documents in each cluster, we employed the Latent Dirichlet Allocation (LDA) [25, 26] approach for topic modeling. LDA represents each scholarly document as a distribution of topics and each topic as a distribution of keywords. In Table 2, we present an example of the keyword list for each topic. Specifically, the keywords listed in Table 2, represented in the “Terms” column, were identified as the most probable terms to be associated with each topic. These keywords were used to define the topic clusters. Specifically, the first three keywords were chosen as the cluster name based on their high probability of being associated with that topic.

#### Train and test split

In this study, we employed a stratified sampling approach to split the data for our classification task of influential level. Specifically, we split the test and train data within each of the categorized datasets (WHO-4 and WHO-30) to ensure that the distribution of classes within the test and train sets is representative of the overall distribution of classes within the categorized data. This approach guarantees that the model was not biased towards any particular class when making predictions. Additionally, we utilized the entire corpus for the uncategorized data and applied a single train and test split across the entire corpus. This approach allows us to evaluate the performance of our models on both categorized and uncategorized data and provides an overall view of the performance of our method. This also allows us to compare the performance of the models when they are trained and tested on both categorized and uncategorized data, which helps us understand the effect of categorization on the performance of our models. The train and test split workflow for the WHO-4 and WHO-30 categorized data is presented in Fig. 6.

#### Data pre-processing

According to the literature review, stopword removal enhances interpretability but has minimal effect on classifier accuracy [18]. We also utilize the substituting stems and features with their separate lemmas. Both methods reduced classifier accuracy. Therefore, they were not employed in the pre-processing stage.

#### Document representation methods

The Term Frequency-Inverse Document Frequency (TF-IDF) weighting method is the most popular document representation method for scholarly documents. The TF-IDF document representation method was designed using extracting uni-grams, bi-grams, and tri-grams from the scholarly document.

The main goal of the BOW (binary) document representation method was to improve interpretability. BOW was defined as a binary incidence matrix based on uni-grams, bi-grams, and tri-grams. Like the TF-IDF document representation, the BOW representation was built using extracted uni-grams, bi-grams, and tri-grams.

The Bidirectional Encoder Representations from Transformers (BERT) was utilized state-of-the-art for the embeddings-based document representation method. As with other document representation techniques, we utilized the same set of WHO corpus and applied it with the BERT Tokenizer. We used the pertained model with twelve hidden layers with twelve attention heads. The weights were the same as the original authors, and English Wikipedia [27, 28] and BooksCorpus corpus [27, 28] used for the pre-training.

#### Machine learning methods (Influential scholarly documents prediction)

For the machine learning experiments, we utilize the random forest [7, 29], linear support vector machine (Linear SVC) [30] and logistic regression [31] classifier as implemented in the scikit-learn^4^, a free software machine learning library for python. For training and testing purposes, we consider each scholarly document’s abstract.

**Table 3:** Overview of input Parameter grid

Machine learning algorithm	Parameter grid
Random Forest	
	$\bullet$ *’max_depth’: 10, 150, 500, 1000*,
	$\bullet$ *’max_features’: 30, 500, 3000*,
	$\bullet$ *’min_samples_leaf’: 1, 10, 100*,
	$\bullet$ *’min_samples_split’: 2, 10, 100*,
	$\bullet$ *’n_estimators’: 10, 100*
Linear Support Vector Machine	
	$\bullet$ *’loss’: ’hinge’*,
	$\bullet$ *’penalty’: ’l2’*,
	$\bullet$ *’alpha’: 1e-3*,
	$\bullet$ *’random_state’: 42*,
	$\bullet$ *’max_iter’: 5*,
	$\bullet$ *’tol’: None*
Logistic Regression	
	$\bullet$ *’n_jobs’: 1*,
	$\bullet$ *’C’: 1*
Neural Network (BERT)	
	$\bullet$ *’batch_size’: 3*,
	$\bullet$ *’lr’: 1e-5*,
	$\bullet$ *’eps’: 1e-8*,
	$\bullet$ *’epochs’: 5*,
	$\bullet$ *’seed_val’: 17*

For the neural network (BERT) experiment utilized an open-source Transformer library [13] from Hugging Face^5^. For our experiment, the BERT model processed input tokens in three steps, and each token is the sum of Token, Segment, and Position embedding. We used Bert-base-uncased [28] for this experiment, which contains 12 Transformer Encoders, 12 attention heads, and 110M parameters. The uncased means it does not differentiate between upper-case and lower-case-related issues. For the pre-trained BERT model, we used “BertTokenizer” [13] from this library because this model had a specific, fixed vocabulary, and the BERT tokenizer had a particular way of handling out-of-vocabulary words. For tokenizing the corpus, we used “batch_encode_plus.” It finds all of the unique words in the corpus and assigns each of them a unique integer. To design the classification task, we utilized the “BertForSequenceClassification” where “from_pretrained” was used to load the pre-trained model and num_labels for the number of labels. We used “AdamW” [13] optimizer as an optimizer from the Huggingface library.

We also utilized different types of hyperparameters to find the best result using different machine-learning methods. We used the k-fold cross-validation from the scikit-learn. It provides us with the cross-validation with grid search hyperparameter optimization via the GridSearchCV^6^ classes, respectively. We used the inner loop of nested cross-validation, where the outer loop defined the training dataset is used as the dataset for the inner loop. We also configure the hyperparameter search to refit a final model with the entire training dataset using the best hyperparameters. As we described before, we utilize nested cross-validation for fine-tuning the hyperparameters. A nested cross-validation is an approach for model hyperparameter optimization that attempts to overcome the problem of overfitting the training dataset. The procedure involves treating model hyperparameter optimization as part of the model itself and evaluating it within the broader k-fold cross-validation^7^ procedure for evaluating models for comparison and selection. A set of different hyperparameters for the different machine learning methods was optimized according to the grid in Table 3.

#### Feature extraction for model explainability

Numerous feature importance approaches were proposed for the random forest, support vector machines, logistic regression, and neural networks (BERT), but many of these methods still need to produce consistent results [32]. In addition, it is possible to compute feature significance for models like the random forest using various model-independent techniques. In this study, Shapley values (SHAP) [33] and LIME [34] were used. These techniques give local feature importance values for a specific test instance instead of the scikit-learn feature-importances method for the random forest, which provides global feature importance scores.

**SHapley Additive exPlanations (SHAP).** Shapley’s notion from game theory advances the SHAP value. A global interpretation was also possible using the SHAP values. Each observation receives a unique set of SHAP values, allowing for local interpretation of the data.

**Local Interpretable Model-agnostic Explanations (LIME).** LIME demonstrates which features values and how they affect a specific prediction. This explanation can only be considered approximate because the LIME model was developed by altering the explained instance by varying the feature values and observing the effects on the prediction of each feature change. The explanation was obtained by replacing the described model locally with an interpretable one.

**Feature importance for Random Forest.** Random forest models were challenging to understand because there were many trees. The trees were sophisticated, and several trees created an impact on choice. However, the random forest learning approach was designed so that producing estimates of feature relevance scores is straightforward [35]. In this research, we utilized random forest feature importance scores using the scikit-learn feature-importances technique^8^, which was based on the mean and standard deviation of accumulation of impurity decreases inside each tree.

### Leveraging the domain-independent Knowledge Graph DBpedia for improved influential paper classification

An annotation tool called DBpedia Spotlight [36] extracts entities from the abstracts. We utilized those entities to identify the DBpedia resources, and the results were filtered based on confidence, support, and similarity score measures. For example, ’antibiotic,’ ’mechanical_ventilation,’ and ’dysbiosis’ were the three different entities that were extracted from the abstract for the scholarly document called “The importance of airway and lung microbiome in the critically ill” [37] (DOI: https://doi.org/10.1186/s13054-020-03219-4). These entities fulfill all the requirements from Table 4 (the parameters used for this experiment). The selected entities were connected to their respective URIs from DBpedia.

**Table 4:** DBpedia Spotlight Parameters

Entities	Similarity score	Support	Confidence
3	0.90	7	0.90

In DBpedia Spotlight, “confidence” measures the likelihood that a text fragment corresponds to a specific DBpedia resource. The confidence value is between 0 and 1, generated by the DBpedia Spotlight’s annotation algorithm. The higher the confidence score, the more likely the text fragment corresponds to the identified DBpedia resource.

In DBpedia Spotlight, “support” is a metric that counts how many times a particular DBpedia resource is mentioned in the annotated text. This metric can be used to determine the popularity or frequency of an entity in the text and can provide additional information on the relevance and context of the annotation. The DBpedia Spotlight API returns the support count and other information, such as the identified resource’s confidence score, surface form, offset, and URI. This information can help evaluate the relevance and quality of the annotation and identify errors or mistakes in the annotation process.

In DBpedia Spotlight, “similarity score measures” are methods used to calculate the similarity between a text fragment and a DBpedia resource, which is used in determining the confidence of the annotation. Similarity Score measures are used to compare the text fragment being annotated to the resources available in the DBpedia KG, and this is done by comparing the surface form of the text fragment with the labels and alternative labels of the resources in the graph and comparing the context of the text fragment with the abstract of the resource.

**Generators.** A KG offers a diverse spectrum of additional features such as specific, unqualified, qualified relation, entity type, etc [38]. In this work, we obtained such features from the DBpedia KG. Then we converted these newly generated features into the form of additional columns. The input was designed to contain at least one column holding URIs to establish connections between the KGs. Such as for ’antibiotic,’ the additional features will be ’Anti-infective_agents’ and ’Bactericides’ using the generators. We only utilized the Direct Type and Unqualified Relation generators for this experiment.

- **Direct type** - Direct types refer to the explicit assignment of a class or category to an entity (using rdf:type) within the graph. This assignment allows for more accurate and efficient querying of the KG and understanding of the entities within it.
- **Unqualified relation** - Unqualified relations refer to edges in the graph that need a more formal qualification, such as a label or a type, which can make it challenging to understand the definition and context of the relationship. This might be a powerful constraint when utilizing DBpedia for particular applications like information extraction and semantic search. Our research focused on identifying and measuring the instances of unqualified relations in the DBpedia knowledge graph and utilizing it for the influential scholarly documents prediction task. As an example, the unqualified connection between the entities “Prague” and “Charles Bridge” in DBpedia would be “There is a connection between Prague and Charles Bridge.” The relationship between these two entities has not provided any additional information.

## Results

### Predicting influential scholarly documents

In this section, we reported on analyzing the scholarly document label classification and the influential scholarly document prediction with different machine learning methods. We also not only considered the influential scholarly documents prediction for the uncategorized data, but our main focus was to predict the influential scholarly documents inside the categorized data (inside each label). First, we analyze the prediction quality for the label classification with the WHO-4 and WHO-30 data. Second, we analyze the quality of the influential scholarly document’s prediction inside categorized and uncategorized data.

For the influential scholarly document prediction, we evaluated the performance of the predictors using the accuracy metric. To calculate these metrics, we utilized the predictors’ average precision, recall, and accuracy for the prediction task of “High” or “Low” influence within the WHO-4 and WHO-30 data. For the WHO-4 data, we calculated the aggregate precision, recall, and accuracy for a predictor with “High” or “Low” influence and divided it by four. Similarly, for the WHO-30 data, we calculated the aggregate precision, recall, and accuracy for a predictor within “High” or “Low” influence and then divided it by thirty. This method allowed us to more accurately represent the predictor’s overall performance for influential scholarly document prediction within the WHO-4 and WHO-30 data.

**Table 5:** Best prediction quality for the scholarly document category classification; WHO = World Health Organization (COVID-19 Global literature on coronavirus disease); LR = Logistic Regression; P = Precision; R = Recall; F1 = F1 score; A = Accuracy; MA = Macro average; WA = Weighted average

Data	ML	Repr.	P	R	F1	A	MA	WA
WHO-4	LR	TF-IDF	0.83	0.84	0.84	0.84	0.84	0.84
WHO-30	LR	TF-IDF	0.92	0.93	0.92	0.93	0.92	0.92

**Table 6:** Best average prediction quality for influential scholarly document classification; WHO = World Health Organization (COVID-19 Global literature on coronavirus disease); RF = Random Forest; P = Precision; R = Recall; F1 = F1 score; A = Accuracy; MA = Macro average; WA = Weighted average

Data	ML	Repr.		P	R	F1	A	MA	WA
WHO-Un	RF	TF-IDF	High	0.75	0.73	0.74	0.74	0.74	0.74
			Low	0.73	0.75	0.74			
WHO-4	RF	TF-IDF	High	0.64	0.43	0.51	0.67	0.67	0.67
			Low	0.69	0.83	0.75			
WHO-30	RF	TF-IDF	High	0.66	0.39	0.47	0.68	0.61	0.65
			Low	0.69	0.85	0.76			

#### Scholarly document category classification

We divided the corpus into 70% training and 30% testing to assess predicted performance. The accuracy was calculated as the number of correct classifications divided by the total number of predictions. We also assessed the F1 score of the methods. However, we excluded this for performance because it was often almost similar (within 2%) to the model’s accuracy.

The best results for the scholarly documents domain or label classification were presented in Table 5. From this experiment, we found that the prediction accuracy was lower than for all BOW representations of the abstracts. Interestingly, the logistic regression method with the TF-IDF document representation method performed better than all other machine learning-based methods. The logistic regression method achieved 0.84; there was a 4% difference between the random forest method trained over TF-IDF-based representation and the logistic regression method with the same document representation method. On the other hand, the embedding-based approach (BERT) is less accurate than the logistic regression method with TF-IDF document representation.

On the other hand, the 30-label classification with WHO-30 data and TF-IDF document representation with logistic regression also got the best accuracy with 0.93. In contrast, the other machine learning methods get less accuracy with the TF-IDF and BOW document representation methods. We got inferior accuracy for the embedding base approach (BERT) than the other document representation methods with different machine learning methods.

All the experiment results for the scholarly documents label classification are publicly available in a GitHub repo with all the machine learning methods.).

#### Influential scholarly document prediction

As before, we divided each cluster from each corpus class into 70% training and 30% testing to assess predicted performance for the influential scholarly document prediction.

We conduct multiple experiments in this direction to determine the influential scholarly document prediction. With the uncategorized data in Table 6, random forest with TF-IDF document representation worked best with 0.74 accuracies for the influential scholarly document prediction. BOW with the random forest, TF-IDF, BOW with logistic regression, and embedding base approach (BERT) got almost the same accuracy as the random forest with TF-IDF document representation.

For the influential scholarly document prediction with the WHO-4 data classifier, the TF-IDF input data table using random forest got 0.67 accuracies, whereas SGD achieves almost similar accuracy. For this, the random forest classifier performs almost similarly to the SGD and logistic regression methods. Also, for the WHO-30 data classifier, the TF-IDF input data table using random forest got 0.68 accuracies. On the other hand, the embedding base approach (BERT) also got similar accuracies as other machine learning methods.

All the experiment results for the scholarly documents label prediction are publicly available in a GitHub repo with all the machine learning methods.

**Table 7:** Best impact of the KG for the scholarly document category classification task with different machine learning methods; WHO = World Health Organization (COVID-19 Global literature on coronavirus disease); LR = Logistic Regression; RF = Random Forest; P = Precision; R = Recall; F1 = F1 score; A = Accuracy; MA = Macro average; WA = Weighted average; D_U_B = DBpedia Unqualified Relation with Bag-of-words; D_D_B = DBpedia Direct Type with Bag-of-words

Data	ML	Repr.	P	R	F1	A	MA	WA
WHO-4	RF	BOW	0.80	0.79	0.79	0.79	0.79	0.79
WHO-4	RF	D_U_B	0.80	0.79	0.79	0.79	0.79	0.79
WHO-4	RF	D_D_B	0.80	0.78	0.78	0.79	0.78	0.79
WHO-30	LR	BOW	0.81	0.87	0.81	0.82	0.82	0.82
WHO-30	LR	D_U_B	0.81	0.87	0.81	0.82	0.82	0.82
WHO-30	LR	D_D_B	0.81	0.87	0.81	0.82	0.82	0.82

**Table 8:** Best impact of the KG for the influential paper classification task (WHO = World Health Organization (COVID-19 Global literature on coronavirus disease); LR = Logistic Regression; RF = Random Forest; P = Precision; R = Recall; F1 = F1 score; A = Accuracy; MA = Macro average; WA = Weighted average; D_U_B = DBpedia Unqualified Relation with Bag-of-words; D_D_B = DBpedia Direct Type with Bag-of-words

Data	ML	Repr.		P	R	F1	A	MA	WA
WHO-Un	RF	BOW	High	0.96	0.58	0.72	0.78	0.77	0.77
			Low	0.70	0.98	0.81			
WHO-Un	RF	D_U_B	High	0.97	0.58	0.72	0.78	0.77	0.77
			Low	0.70	0.98	0.82			
WHO-Un	RF	D_D_B	High	0.96	0.58	0.72	0.78	0.77	0.77
			Low	0.70	0.98	0.81			
WHO-4	LR	BOW	High	0.54	0.54	0.54	0.51	0.51	0.51
			Low	0.51	0.44	0.47			
WHO-4	LR	D_U_B	High	0.59	0.65	0.62	0.66	0.66	0.65
			Low	0.62	0.50	0.54			
WHO-4	LR	D_D_B	High	0.54	0.58	0.56	0.62	0.61	0.61
			Low	0.50	0.45	0.47			
WHO-30	LR	BOW	High	0.62	0.65	0.63	0.64	0.64	0.64
			Low	0.66	0.65	0.64			
WHO-30	LR	D_U_B	High	0.70	0.80	0.73	0.71	0.69	0.69
			Low	0.66	0.63	0.64			
WHO-30	LR	D_D_B	High	0.65	0.75	0.69	0.67	0.65	0.66
			Low	0.70	0.59	0.62			

#### Differences in influential scholarly document classification for the categorized and uncategorized datasets

**Fig. 7:**
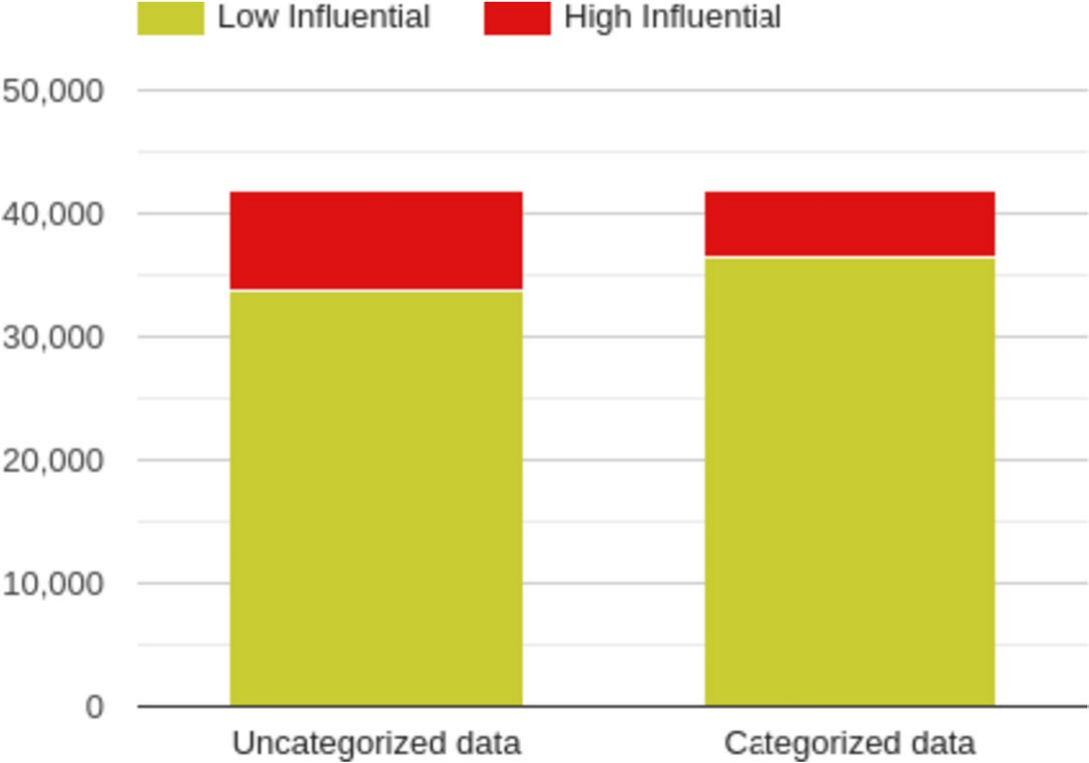
Difference between the COVID-19-related influential (High and Low-level) papers (From the year 2022) with un-categorized and four-level (WHO) categorization

**Fig. 8:**
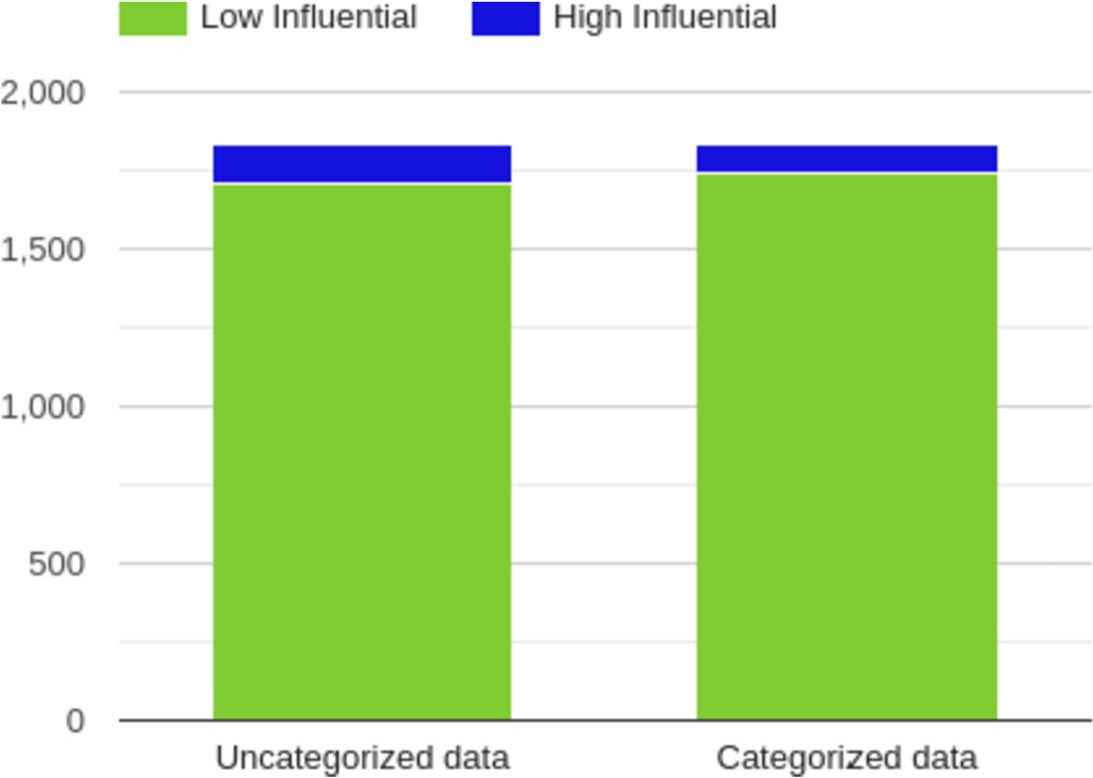
Difference between the COVID-19-related influential (High and Low-level) papers (From the year 2023) with un-categorized and four-level (WHO) categorization

For most influential scholarly documents prediction [10], researchers only utilized the un-categorized scholarly documents. In Fig 7, we found that the 33692 scholarly documents published in 2022 were classified as “Low” influential scholarly documents by the random forest method, which was trained by the uncategorized data. At the same time, 36502 scholarly documents were classified as “Low” influential scholarly documents by the random forest method that was trained by WHO-4 data. On the other hand, 8220 scholarly documents published in 2022 were classified as “High” influential scholarly documents by the random forest method, which was trained by the uncategorized data. In comparison, 5418 scholarly documents were classified as “High” influential by the random forest method that was trained by the WHO-4. In Fig 8, we found that the 1711 scholarly documents published in 2023 were classified as “Low” influential scholarly documents by the random forest method, which was trained by the uncategorized data. At the same time, 1743 scholarly documents were selected as “Low” influential scholarly documents by the random forest method that was trained by the WHO-4 data. In the future, we also plan to verify these influential scholarly documents’ differences by domain experts.

**Fig. 9:**
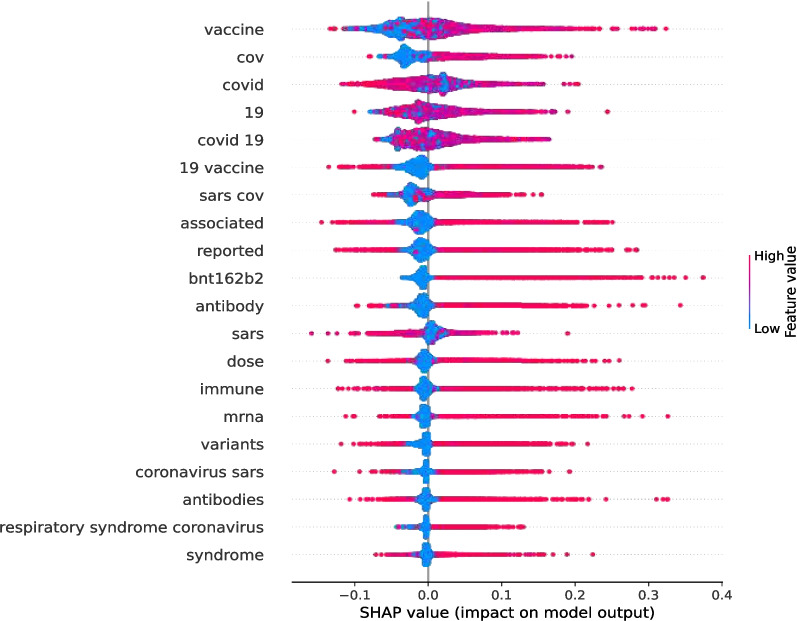
SHAP plot (Vaccines) for an example abstract

**Fig. 10:**
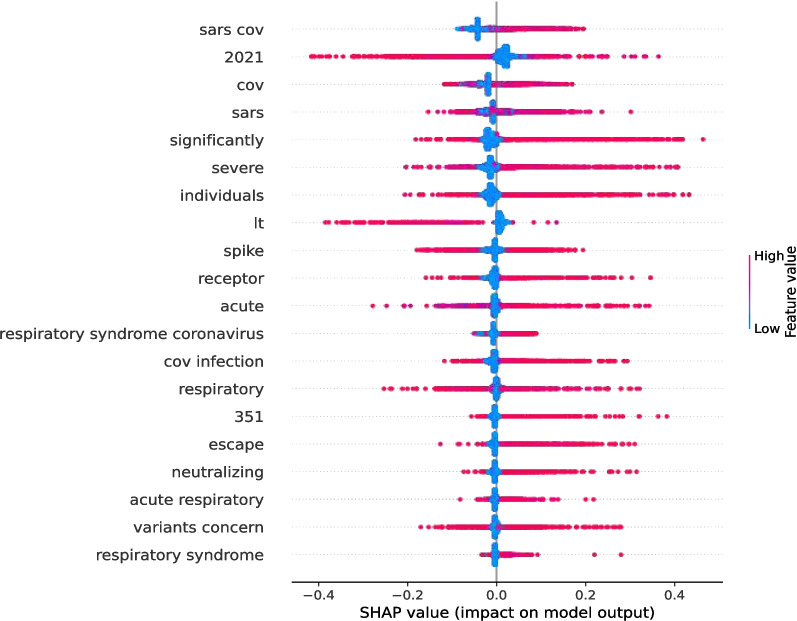
SHAP plot (Variants) for an example abstract

**Fig. 11:**
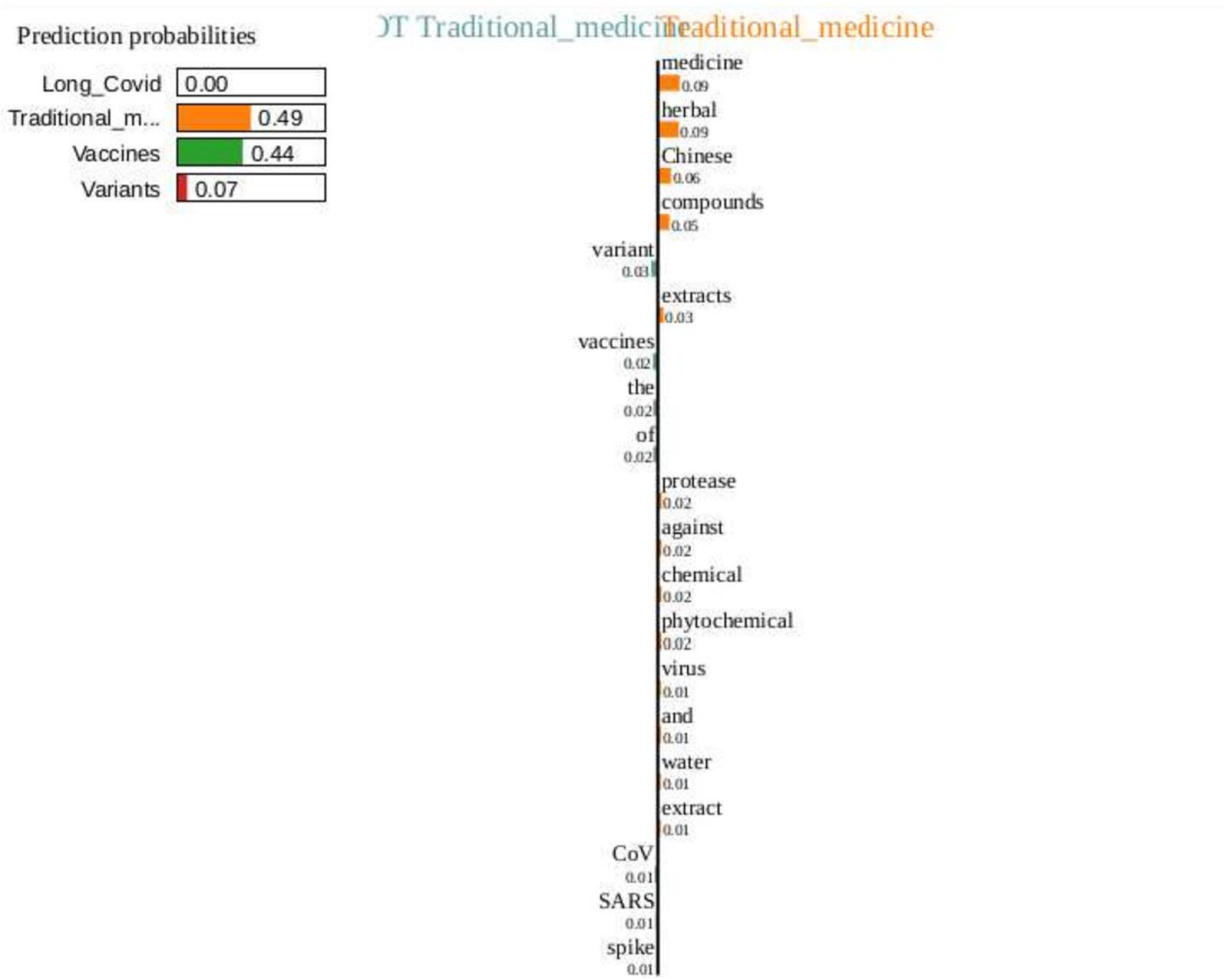
LIME plot for an example abstract

### Impact of the domain-independent knowledge graph (DBpedia) for influential scholarly document prediction

Because of the low response from DBpedia, for this experiment, we considered 5000 randomly selected scholarly documents from the WHO dataset. As before, we also divided the data into 70% for training and 30% for testing to assess predicted performance. The dataset was imbalanced, and we utilized the oversampling method from SMOTE [39]. The accuracy of the results was assessed by dividing the number of correctly predicted observations by the total number of predictions. In addition to accuracy, the F1 score was also considered for this experiment. The baseline system is BOW with different machine learning methods.

Table 7 present the average accuracy for the scholarly documents categorization task with the domain-independent KG. We did not find any impact in utilizing the domain-independent knowledge base for a scholarly document category classification task. Then an enhanced version of the document representation method gets the same accuracy as the BOW document representation method with different machine learning methods.

For the influential scholarly document prediction, the DBpedia unqualified relation with bag-of-words (D_U_B) worked well with LR for the WHO-4 data. For the WHO-30 data, the DBpedia unqualified relation with bag-of-words (D_U_B) works well with the LR. On the other hand, the domain-independent KG had no significant impact on the influential scholarly document prediction task with uncategorized data. The average evaluation metrics evaluate the overall performance - both positive classes (highly cited scholarly documents) and negative ones (lowly cited scholarly documents). Using the domain-independent KG, we achieved better accuracy in estimating incredibly highly influential versus lowly influential for the WHO-4 and WHO-30 data - which is surprising. We plan to investigate this surprising improvement in more detail in the future (Table 8).

## Model explainability

We utilize LIME, SHAP, and univariate statistics to describe the feature importance locally (for each scholarly document’s abstracts). Feature importance values were used to analyze the scholarly document’s label classification with the Random Forest methods (Workflow for the Fig. 1) using the mean and standard deviation. Table 9 shows the top features by relevance computed using the mean and standard deviation of accumulation of impurity decrease inside each tree technique for the TF-IDF and BOW input data table.

SHAP can detect the direction of feature significance in the same way as the mean and standard deviation techniques can. A SHAP plot in Figs. 9 and 10 illustrates the significance of specific words for WHO-4 data label “Vaccines” and TF-IDF with WHO-4 data label “Variants.” The colour represents the feature value, whereas the red colour shows greater values than the values in blue. Vaccine, cov, covid, sars cov are examples of features that contributed to the abstract being classified into the influential category, as the corresponding SHAP value was mostly positive when the feature value (TF-IDF score) was high. The word “vaccine” could have been expected to be important as this result was obtained for the *Vaccines* category (WHO-4). On the other hand, sars cov, sars, severe, escape, and many more were important features for the scholarly document abstract using TF-IDF for this scholarly document influential level prediction task inside the *Variants* category with WHO-4 data. An interesting, important feature among those identified for the Variants category with WHO-4 data is “escape”.

We also included the LIME figure in Fig. 11 to explain the influential level prediction of the Random Forest model for a sample scholarly document based on its abstract. The model predicts the abstract of a scholarly document as “High” influential, with some of the features contributing to the scholarly documents belonging to the highly influential category being “sars,” “convalescents,” and “vaccinated”. In the future, we plan for a more in-depth analysis of model interpretability. We plan to find the important feature for each categorized dataset for the influential scholarly document classification with the WHO-4 and WHO-30 data.

**Table 9:** Top Features (Mean and Std. Dev.) for Categorized Data

TF-IDF (WHO-4)	Imp.	BOW (WHO-4)	Imp.	TF-IDF (WHO-30)	Imp.	BOW (WHO-30)	Imp.
variants	0.013	vaccine	0.013	posted listserv copyright	0.002	children	0.019
vaccine	0.011	vaccination	0.012	students	0.002	vaccina-tion	0.017
medicine	0.006	variants	0.008	individual use	0.002	students	0.016
vaccination	0.005	vaccines	0.007	thromboc-ytopenia	0.001	omicron	0.014
vaccines	0.005	studies	0.007	se	0.001	vaccine	0.013
variant	0.005	article	0.007	antibodies	0.001	symptoms	0.010
herbal	0.004	sarscov2	0.006	children	0.001	model	0.007
medicinal	0.004	variant	0.006	vaccine	0.001	sars	0.006
treatment	0.003	patients	0.006	docking	0.001	mental	0.006
chinese	0.003	pandemic	0.005	antibody	0.001	thromboc-ytopenia	0.006
antiviral	0.003	infection	0.005	thrombotic	0.001	95 ci	0.006
traditional chinese medicine	0.003	covid19	0.005	igg	0.001	health	0.006
traditional	0.003	different	0.004	cov	0.001	sars cov	0.005
covid19 vaccination	0.003	conclusions	0.004	abridged	0.001	variant	0.005
covid19 vaccine	0.002	disease	0.004	mental health	0.001	igg	0.005

### Concerning the domain-independent Knowledge Graph (DBpedia)

As part of the experiment, we also obtained the importance of variables from combined models - BOW and KG. However, in the combined method, among the top features, variables from the KG never moved above important variables from BOW. Among the top variables from the BOW model, we can see essential terms from abstracts referring to coronavirus disease - e.g., covid, covid - 19, cov, sars cov, virus. In particular, ’cov’ and ’virus’ also came out among the top indicators using the Shapley values in our previous research  [10], which show that these terms lead to an increase in the probability of a scholarly document being highly cited. Another more easily justified important variable is the word ’expression.’ Gene expression often comes up in combination with the coronavirus in researching what levels of gene expression in humans can lead to a higher susceptibility to getting sick with the coronavirus. Although the KG was not domain specific, in some cases, it showed association and importance with the disease and virus (Animal_virology, Diseases_of_liver). A noisy feature in a classification or prediction task refers to a feature introduced from external sources that can negatively impact the classifier. In this case, the domain-independent KG also provides features not directly related to the classified data and can introduce additional, potentially irrelevant information. Noisy features can lead to overfitting or bias in the classifier, even reducing its overall performance.

**Fig. 12:**
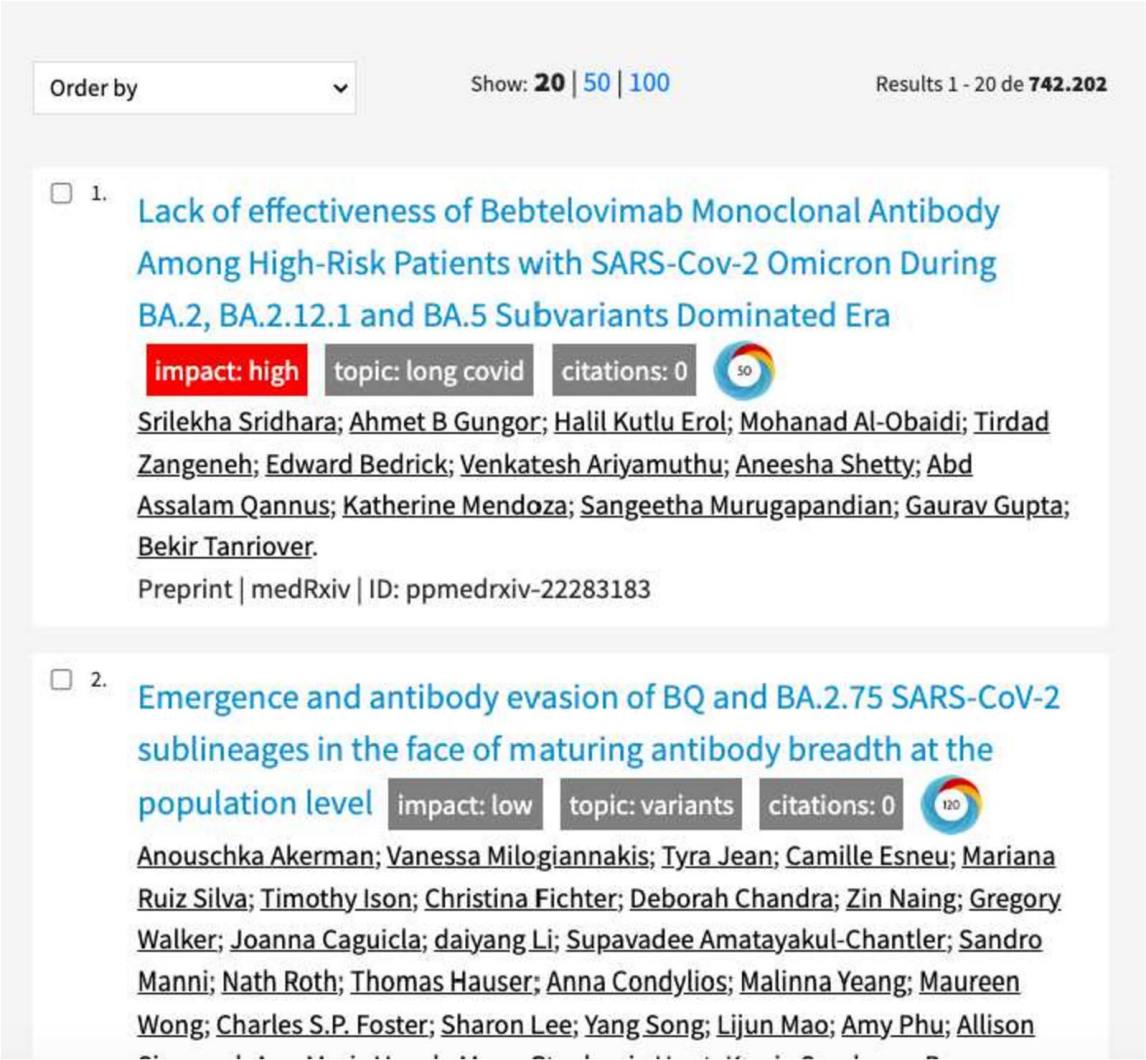
Screenshot of the implemented browser extension within the WHO COVID-19 Research Database website

## Implementation

To demonstrate the usefulness of our approach in a real-life setting, we implemented our model as a service to create a browser extension. Since our model can predict how influential a paper can become, it provides value to users exploring papers and seeking specific information. The browser extension has been designed to provide additional information to users exploring COVID-19-related research, explicitly using the WHO COVID-19 Research Database. Instead of creating a new user interface to explore the COVID-19 dataset, we decided to extend the existing well-established exploration interface of the WHO. Therefore, we created a browser extension activated when the browser’s COVID-19 research database interface is visited. Figure 12 depicts a screenshot of the activated extension. The extension tries to find all paper titles on the displayed page and appends additional labels to the title. The following information is appended to paper titles: the predicted impact (either low or high), topics, citation count, and an Altmetric [40] indicator.

The backend service of the browser extension has been written in Python and provides a REST API consumed by the extension. The service accepts a paper title as input and returns the DOI, influential level, and topics as output. In turn, the DOI fetches the citations and Altmetric indicator. The browser extension is explicitly developed for the Google Chrome browser and written in Javascript. The source code is available online 9.

## Strengths and limitations of this work

### Strengths of this work

The influential scholarly document prediction task serves the scientific community. In particular, it provides the following benefits:

- **Efficient prioritization:** Researchers such as the ones focusing on the COVID-19 domain can quickly identify scholarly documents with a higher impact [10], enabling them to prioritize their reading effectively [41] [42].
- **Focused exploration:** In areas with vast amounts of new scholarly documents, such as the COVID-19 pandemic or infectious diseases in general, prediction helps researchers to access influential works or quality assessment, thus facilitating targeted exploration efficiently [43].
- **Resource allocation:** Decision-makers can allocate limited resources, like funding and time, more judiciously by recognizing influential work that warrants greater attention.
- **Timely updates:** Researchers can stay informed about new domain-related discoveries and influential research developments in real-time by identifying and accessing highly influential scholarly documents [44].
- **Trend identification:** Predicting influence aids in recognizing emerging trends and hot topics within a field such as COVID-19, assisting researchers in staying at the forefront of their discipline [45] [46] [47].
- **Citizen science:** During disaster periods such as pandemics or natural calamities, citizens, governing bodies, funders, etc. start seeking relevant information to stay informed. During such disasters, which may last up to several years, research that starts accruing influence should be highlighted for the sake of citizen science. Systems such as the ones we propose can predict the potential to be highly influential based on contextual information. For shorter and actual disasters there is less opportunity for scientometric indicators to accrue for works tackling these and automatically spotlighting relevant potential influential research is highly relevant for solving the societal challenge and citizen science [48] [49].

Influential scholarly document prediction enhances the utility of domain-related scholarly documents, streamlining the exploration of scholarly documents and contributing to the advancement of scientific knowledge. Note we do not propose our method as a replacement for predicting the most relevant work on a certain topic. It is merely an alternative way to facilitate prioritization in the publication flood and let the researchers determine the ultimate usefulness. Our method might be particularly beneficial during timely societal challenges to quickly access relevant research results.

### Limitations of this work

Our previous research [10] was the first attempt to identify influential scholarly documents using the contents with different machine learning methods. We tried to overcome the hypothetical limitations, but our research suffers from citation count limitations. Opencitation citation count is comparable to commercial services source for the citations in the scholarly knowledge graph domain [10], with multiple scholarly knowledge graphs currently (such as Open Research Knowledge Graph (ORKG)) using it in their research [50]. However, for some uses, the quality of citation data in Opencitation was limiting [51]. As an example, the scholarly document called “Factors associated with COVID-19-related death using OpenSAFELY” [52] citation count was 2984, where we did not find any citation count in the Opencitation (Version 1.3.0). Also it is important to contextualize the scope and applicability of our research findings. While our decision to collect data within a relatively short timeframe and concentrate solely on COVID-19-related scholarly documents was motivated by the urgency of the pandemic, it does bring about specific limitations. Firstly, the time-bound nature of the dataset may not fully capture the enduring influence of scholarly works that evolve and gain prominence over an extended period. Secondly, the study’s only focus on COVID-19 restricts the generalizability of our findings to other research domains. However, it is imperative to underscore that our approach holds particular relevance in disaster response scenarios, where immediate decision-making is critical. It offers a valuable means of predicting influential scholarly documents in real-time, complementing traditional scientometric approaches that assess impact over a longer duration. Nevertheless, these limitations underscore the need for future research endeavors to address broader research domains and encompass a more extensive timeframe to provide a comprehensive understanding of scholarly influence dynamics across diverse contexts.

## Conclusions and future work

Our work categorizes the WHO COVID-19-related scholarly documents into high and low-influential classes with a new dimension regarding the labels in a corpus. The proposed pipeline can thus help to filter the influential WHO scholarly documents dealing with COVID-19-related issues from a massive amount of scholarly documents. The impact of incorporating categorical information on the prediction of influential scholarly documents has been investigated in this study. The performance of various document representation techniques combined with different machine learning methods was evaluated for classifying scholarly documents into four and thirty different categories (WHO-4 and WHO-30 data). The study results indicate that logistic regression, in combination with TF-IDF document representation, performed best for classifying scholarly documents category into WHO-4 and WHO-30. It was found that the use of random forest in combination with TF-IDF document representation yielded optimal results for the prediction of influential scholarly documents within both uncategorized and categorized data. For this experiment, we run each machine learning method separately for each category in the categorized data (WHO-4 and WHO-30) to find the effect of using a categorized versus uncategorized corpus on the influential scholarly documents prediction task. However, the study results indicate that the uncategorized corpus performed better in the prediction task than the categorized corpus. The main reason for this, we consider the average accuracy for the categorized corpus, and all the categories in the categorized corpus did not get outstanding results. A possible reason is that the training data for the individual categories in WHO-4 contained 7.5 times more instances, thus allowing the predictors to fit the data better. In the future, we plan to investigate more why the uncategorized corpus performed better and whether the finding is generalizable to other scholarly datasets and machine-learning approaches. In this experiment, we also utilize domain-independent KG (such as DBpedia) to improve the accuracy of the influential scholarly document prediction task by the document representation enhanced with a domain-independent KG. However, it also provides a large number of irrelevant features. In the next step, we plan to apply different rule mining methods to find the different patterns from the highly influential scholarly documents from each label in WHO. On the other hand, in the future, we plan to use different text mining methods with the COVID-19-related scholarly documents for the question-answering system or recommend scholarly documents for individual queries.

## Data Availability

The research has been conducted only with openly available software packages, and prototypes are publicly available in API (https://gitlab.com/rabby2186/who-influential-research-paper-classification) and browser extension (https://github.com/aoelen/who-platform-extension) from the authors.

## Abbreviations

KG: Knowledge Graph
COVID-19: Coronavirus Disease 2019
ML: Machine Learning
TF-IDF: Term Frequency-Inverse Document Frequency
WHO: World Health Organization
BOW: Bag of Words
BERT: Bidirectional Encoder Representations from Transformers
ORKG: Open Research Knowledge Graph
HIV: Human Immunodeficiency Virus
SARS: Severe Acute Respiratory Syndrome
RF: Random Forest
LR: Linear Regression
SHAP: Shapley Additive Explanations
LIME: Local Interpretable Model-agnostic Explanations
LDA: Latent Dirichlet Allocation
PCA: Principal Component Analysis
P: Precision
R: Recall
F1: F1 Score
A: Accuracy
MA: Macro Average
WA: Weighted Average
D_U_B: DBpedia Unqualified Relation with Bag-of-words
D_D_B: DBpedia Direct Type with Bag-of-words
DOI: Digital Object Identifier
RQ: Research Question

## Footnotes

1 https://search.bvsalud.org/global-literature-on-novel-coronavirus-2019-ncov/?lang=en

2 https://opencitations.net/

3 https://scikit-learn.org/stable/modules/generated/sklearn.decomposition.PCA.html

4 https://scikit-learn.org/stable/

5 https://huggingface.co/docs/transformers/model_doc/bert

6 https://scikit-learn.org/stable/modules/generated/sklearn.model_selection.GridSearchCV.html

7 https://scikit-learn.org/stable/modules/generated/sklearn.model_selection.KFold.html

8 https://scikit-learn.org/stable/auto_examples/ensemble/plot_forest_importances.html

9 https://github.com/aoelen/who-platform-extension
